# Multiple signaling functions of song in a polymorphic species with alternative reproductive strategies

**DOI:** 10.1002/ece3.3702

**Published:** 2017-12-27

**Authors:** Melissa L. Grunst, Andrea S. Grunst, Vince A. Formica, Rusty A. Gonser, Elaina M. Tuttle

**Affiliations:** ^1^ Department of Biology Indiana State University Terre Haute IN USA; ^2^ Department of Biology Swarthmore College Swarthmore PA USA

**Keywords:** individual identity, multiple messages, polymorphism, sexual selection, signaling, song, species identity, white‐throated sparrow

## Abstract

Vocal traits can be sexually selected to reflect male quality, but may also evolve to serve additional signaling functions. We used a long‐term dataset to examine the signaling potential of song in dimorphic white‐throated sparrows (*Zonotrichia albicollis*). We investigated whether song conveys multifaceted information about the vocalizing individual, including fitness, species identity, individual identity, and morph. We also evaluated whether song traits correlate differently with fitness in the two morphs, as the more promiscuous strategy of white, relative to tan, morph males might impose stronger sexual selection. Males with high song rates achieved higher lifetime reproductive success, and this pattern was driven by white morph males. In addition, males that sang songs with many notes survived longer, but this pattern was less robust. Thus, song traits reflect differences in fitness and may more strongly affect fitness in the white morph. Song frequency was unrelated to fitness, body size, or morph, but was individual specific and could signal individual identity. Songs of the two morphs displayed similar frequency ratios and bandwidths. However, tan morph males sang songs with longer first notes, fewer notes, and higher variability. Thus, song could be used in morph discrimination. Variation in frequency ratios between notes was low and could function in conspecific recognition, but pitch change dynamics did differ between four different song types observed. Our results support a multiple messages model for white‐throated sparrow song, in which different song traits communicate discrete information about the vocalizing individual.

## 1 INTRODUCTION

Vocal phenotypes, including song in passerine birds, may evolve through more than one selective mechanism, such that the information content of song is multidimensional (Botero et al., 2009; Gil & Gahr, 2002; Rivera‐Gutierrez, Pinxten, & Eens, 2010). Sexual selection is a primary mechanism that influences the evolution of song traits. Sexually selected song traits serve as auditory ornaments that reflect variation in male quality and fitness (Ballentine, 2009; Beecher, Campbell, & Nordby, 2000; Botero et al., 2009; Byers & Kroodsma, 2009; Catchpole & Slater, 2008; Nemeth, Kempenaers, Matessi, & Brumm, 2012; Reid et al., 2005; Rivera‐Gutierrez et al., 2010). However, song also plays other important functions, including signaling species and individual identity (Hurly, Ratcliffe, Weary, & Weisman, 1992; Hurly, Ratcliffe, Weisman, & Johnsrude, 1991). How the relative importance of different signaling functions of song varies with male reproductive strategies and social dynamics remains poorly understood. Moreover, whether distinct aspects of the vocal phenotype play different signaling roles is rarely disentangled, as most studies focus on only one signaling function (e.g., sexual signaling).

The sexual signaling function of song might vary between species, or even within species, depending on male reproductive strategy (Garamszegi & Møller, 2004; Greig, Price, & Pruett‐Jones, 2013). In particular, in polygamous species, sexual selection often promotes the evolution of sexually selected traits (Webster, Tarvin, Tuttle, & Pruett‐Jones, 2007; Yezerinac, Weatherhead, & Boag, 1995), including song traits (Shutler & Weatherhead, 1990; but see Sousa & Westneat, 2013). On the other hand, in species where paternal care is essential and polygamy levels are lower, song traits, such as the complexity or rate of vocalizations, might be less commonly employed in sexual signaling. Recent meta‐analyses that controlled for the effect of phylogeny failed to support the hypothesis that the correlation between song complexity and fitness increases with the level of polygyny (Garamszegi & Møller, 2004; Soma & Garamszegi, 2011). Thus, the relationship between polygyny and sexual selection on song remains equivocal. However, across species, different song traits (e.g., performance traits vs. song complexity) could be the target of sexual selection, complicating attempts to use interspecific comparisons to evaluate the relationship between polygyny levels and sexual selection on song (Cardoso & Hu, 2011).

Indeed, song is a complex phenotype. Thus, the extent to which song traits act as sexual ornaments and correlate with fitness could vary not only between species, but also between song traits within a species (Gil & Gahr, 2002). Song traits that entail physiological costs, such as singing rate, or are sensitive to environmental stressors, such as repertoire size, have the potential to indicate individual quality and serve as sexual signals (Murphy, Sexton, Dolan, & Redmond, 2008; Pfaff, Zanette, MacDougall‐Shackleton, & MacDougall‐Shackleton, 2007; Spencer, Buchanan, Goldsmith, & Catchpole, 2004; Welling, Rytkönen, Koivula, & Orell, 1997). On the other hand, species identity traits are predicted to entail few costs, are subject to stabilizing selection, and should be relatively stereotyped and show low variability within populations (Becker, 1982; Falls, 1982; Hurly, Ratcliffe, & Weisman, 1990; Hurly et al., 1991; Lambrechts, 1995; Nelson, 1988). Signals of individual identity are also predicted to be relatively inexpensive, arise through negative frequency‐dependent selection favoring distinctiveness, and are especially likely to occur in species with complex social networks (Dale, Lank, & Reeve, 2001; Lank & Dale, 2001; Tibbetts & Dale, 2007). Traits indicative of individual identity have been found across species and behavioral contexts, including neighbor–stranger discrimination, parent–offspring interactions, and dominance hierarchies, and may consist of visual, vocal, or olfactory cues (Beecher, 1982; Dale et al., 2001; Insley, 2000; Tibbetts, 2002; Tibbetts & Dale, 2007).

Song traits might also evolve as a signal or manifestation of alternative reproductive strategies, behavioral types, or morphs. Associations between color polymorphisms and alternative reproductive strategies are well documented (Küpper et al., 2016; Lamichhaney et al., 2016; Pryke & Griffith, 2006, 2007; Sinervo & Lively, 1996). In contrast, examples of polymorphic song traits are rare, but morph‐specific song traits in medium ground finches (*Geospiza fortis*) promote assortative mating (Podos, 2010). In addition to facilitating pairings between genetically compatible individuals, signaling morph identity and behavioral strategy could help to reduce aggressive encounters (Martín & Forsman, 1999), especially where aggressive responses vary with an individual's morph identity (Horton, Hauber, & Maney, 2012). For example, in Australian painted dragons (*Ctenophorus pictus*), experimentally painting males to resemble a different morph prolonged aggressive encounters by 30% (Healey, Uller, & Olsson, 2007). Studies that simultaneously investigate multiple signaling functions of song are needed to clearly elucidate the selective pressures acting on complex vocal phenotypes.

**Figure 1:**
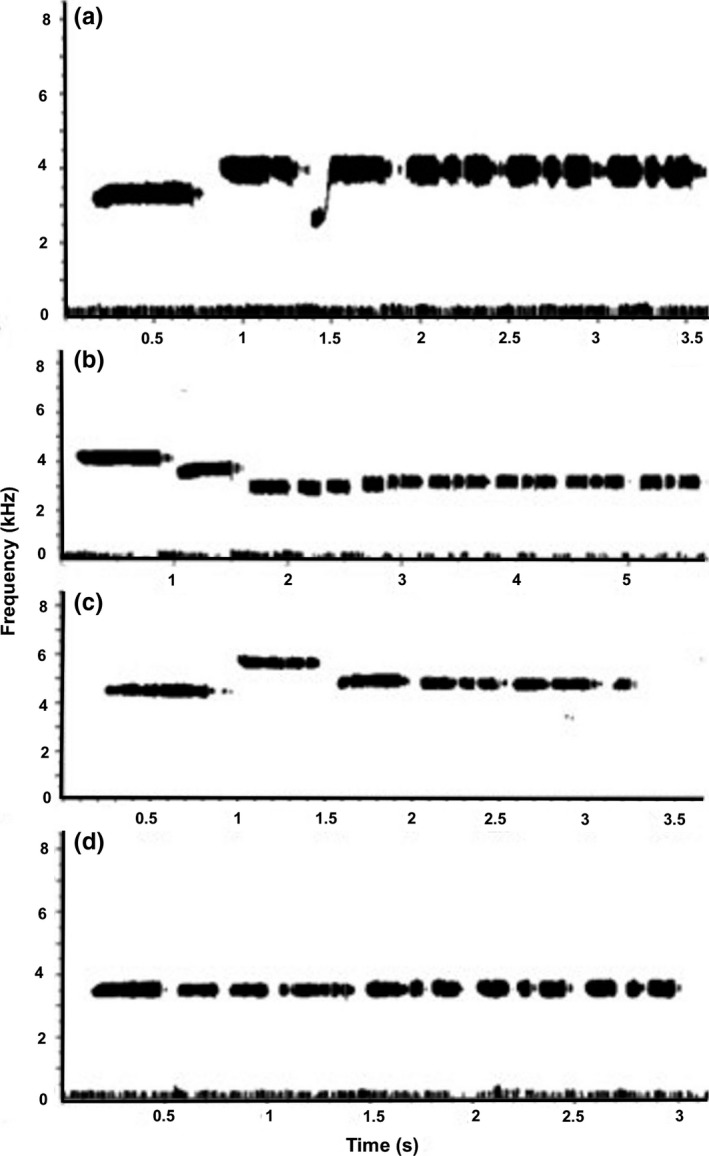
Male white‐throated sparrows at Cranberry Lake displayed four distinct song types: (a) ascending, (b) descending, (c) ascending and then descending, and (d) monotone. The third note in the ascending song corresponds to Borror and Gunn's (1965) U note. All of the song types contain at least one three‐part triplet

We examined multiple signaling functions of song in the dimorphic white‐throated sparrow (*Zonotrichia albicollis*). First, we examined the relationship between reproductive strategy and the sexual signaling function of song, which may be easier to detect when comparing morphs within a species than when making interspecific comparisons. In the white‐throated sparrow, males of two genetically differentiated morphs display distinct reproductive strategies, but very similar ecologies (Taylor & Campagna, 2016; Thorneycroft, 1966, 1975; Tuttle et al., 2016). Males of both morphs are territorial, but white morph males exhibit higher aggressiveness and rates of extra‐pair mating than tan morph males, which are largely monogamous and invest in mate‐guarding and paternal care (Falls & Kopachena, 2010; Formica & Tuttle, 2009; Horton, Moore, & Maney, 2014; Horton et al., 2012; Kopachena & Falls, 1993a,1993b; Tuttle, 1993, 2003). In addition, white morph males exhibit exaggerated visual and vocal signals, with brighter plumage coloration and higher song rates than tan morph males. Based on these differences in reproductive strategy (Tuttle, 2003), we predicted stronger correlations between song traits and fitness, consistent with stronger sexual selection on song traits, in white morph males. White‐throated sparrow song consists of serially produced, ascending or descending high‐pitched whistles, often ending in a series of three‐part notes known as triplets (Figure 1; Borror & Gunn, 1965). Males usually sing a single song type (Borror & Gunn, 1965). Thus, song complexity is unlikely to be the target of sexual selection. However, performance traits such as the frequency and range of the vocalization (Ballentine, Hyman, & Nowicki, 2004; Christie, Mennill, & Ratcliffe, 2004), song rate (Murphy et al., 2008; Welling et al., 1997), or singing consistency (Botero et al., 2009; Byers, 2007; Taff et al., 2012) could play a sexual signaling role.

We also investigated the role of white‐throated sparrow song in signaling individual and species identity. With respect to individual identity, past studies have established that white‐throated sparrow songs are individually distinctive (Brooks & Falls, 1975a), and manipulated songs to determine which song characteristics allow for neighbor–stranger discrimination (Brooks & Falls, 1975b; Hurly et al., 1991). We explored which song traits most strongly differentiate individuals on the population level, which has not been previously quantified. With respect to species identity, past research in the white‐throated sparrow suggests that the frequency ratio between notes is an invariant species identity trait, whereas the absolute frequency range (bandwidth) varies between individuals (Hurly et al., 1991, 1992). However, conclusions regarding the magnitude and relative variability of these traits in the white‐throated sparrow rely on information from a single population. Furthermore, previous work has focused on the more vocal white morph (Falls, 1988; Hurly et al., 1991) or has not specified the morph of the singer (Borror & Gunn, 1965). Thus, whether song frequency characteristics are consistent across morphs warrants further investigation.

Indeed, morph‐specific song traits could allow individuals to use song to determine the morph of the singer. We used a relatively large sample of both white and tan morph males to explicitly test this hypothesis. Morph distinctive songs could allow tan morph males to avoid intense aggression from white morph males (Horton et al., 2012), and facilitate adaptive pairing patterns, as white‐throated sparrows pair disassortatively by morph (Falls & Kopachena, 2010; Tuttle, 1993, 2003). Although plumage coloration plays a primary role in distinguishing the morphs, males of the two morphs show some overlap in coloration (Rathbun et al., 2014). Song could help females and males assess male morph in these ambiguous cases and in cases where the singer cannot be seen. Individuals homozygous for the morph‐determining chromosome (ZAL2^m^) occur at extremely low frequencies within populations, suggesting homozygote disadvantage, and a disadvantage of assortative pairing (Thorneycroft, 1975; Tuttle et al., 2016). Thus, selection might favor individuals that signal their morph when singing (Podos, 2010).

Our research made use of an intensive long‐term study on white‐throated sparrows, which allowed recording of known identity males of both morphs and collection of lifetime fitness data. Our findings build on classical work on the song of the white‐throated sparrow and offer new insight into the evolution of song as a multidimensional vocal signal.

## 2 METHODS

### 2.1 Study system and population monitoring

We studied white‐throated sparrows breeding near Cranberry Lake Biological Station (State University of New York College of Environmental Science and Forestry; 44.15N, 74.78W) between 1998 and 2015. We conducted all methods in accordance with rigorous ethical and legal standards. All research was in compliance with the current laws of New York State, the State of Indiana, and the U.S. Federal Government. Procedures were additionally approved by Indiana State University's Institutional Animal Care and Use Committee (protocols 562158‐1 and 562192‐1).

Each season, we monitored breeding pairs to determine territory boundaries and locate nests. We banded individuals with a Fish and Wildlife Service aluminum band and a unique color combination (Master Banding Permit 22296 to E. M. Tuttle). Standard measurements were taken at the time of capture, including tarsus length (±0.01 mm), which we used as a stable metric of body size. Birds were monitored across their entire reproductive lifespan. Our sample included 97 males (62 white and 35 tan). Multiple recordings were obtained for 14 males, and we had data on reproductive parameters for 75 (55 white and 20 tan) males. Sample sizes are reduced for some analyses due to missing data, so we indicate sample sizes for all statistical tests. All but seven males were banded. We used unbanded males only when determining the distribution of song types within the population. The greater proportion of white morph individuals in our sample reflects higher singing rates of white morph males, which facilitates ease of recording.

### 2.2 Recording methods

We used a TASCAM DA‐P1 portable DAT recorder and a Sennheiser ME66 Short Shotgun Capsule to record songs. We identified banded males via color bands during the recording session. Unbanded males were confirmed to be unbanded during the recording session and were identified based on song type, location within a known territory, and morph. We were not able to record data blind, because our study depended on identifying focal individuals in the field. In all years (1998 to 2015), we recorded males between May 14 and August 3, and between 6:15 and 14:52 eastern daylight time. Recordings ranged in length from 2 to 5 min (mean ± *SE*: 4.41 ± 0.28). To efficiently record song, we elicited singing for 57/97 males using playback from multiple (*N* = 8) deceased white morph males. Song was projected using an iPod (Apple, CA, USA) and EGO speakers (amplifier output: 3 W × 2 (RMS); frequency range: 50–20,000 Hz) at a rate that roughly mimics natural singing rates (~5–10 s between songs). We recorded unsolicited singing for the remaining (40/97) individuals. For unsolicited recordings, we did not identify the intended receiver. However, there is no reason to suspect that differences in receivers biased our results. We tested for effects of date, time, and playback use in statistical analyses.

### 2.3 Analyzing recordings

We visualized recordings using Raven Pro version 1.5 (Cornell Lab of Ornithology). We digitized DAT tapes with a sampling rate of 44,100 Hz and sample size of 24 bits. Spectrogram parameters gave a frequency resolution of 172 Hz and a temporal resolution of 2.9 ms (Hann window, overlap = 50%). For each male, we selected all songs produced during the recording. Selections precisely reflected vocalization length, which we calculated using Raven's delta time option. For the five songs with the most notes, we selected each note, recorded the number of notes, and used Raven to calculate note frequency. We calculated the bandwidth (absolute value of the frequency difference between notes) and frequency ratio (ratio between the frequencies of adjacent notes) between both the first and second and second and third notes, and the deviance in the frequency ratio from the population mean. When calculating deviance in the frequency ratio, we used the ratio between the first and second notes for ascending songs, and the ratio between the second and third notes for descending songs. These ratios measure the major frequency shifts in these song types and are proposed to serve as a species identity trait (Hurly et al., 1991). For songs that first ascended and then descended in frequency, we used the frequency ratio that was closest to the mean for the other two song types. We counted the number of notes for all songs produced during the recording interval, and averaged the number of notes to derive a metric of average note number. In addition, we observed that birds sometimes produced very long songs by adding extra three‐part triplets to the ends of their songs (see Figure 1). Thus, for the five songs with selected notes, we counted the number of triplets produced at the end of the song and recorded the maximum number of triplets produced over 3. The maximum number of triplets produced over 3 serves as a metric of a bird's capacity to produce very long songs. Finally, we calculated two metrics of variability in each male's songs. First, as a metric of variability in the frequency of songs, we used the five songs with selected notes and calculated the coefficient of variation for the frequency of the first, second, and third notes, high and low frequency, and bandwidth. We averaged these coefficients of variation to derive a single metric of variability in the frequency of notes produced. Second, as a metric of variability in the length of vocalizations, we calculated the coefficient of variation for the number of notes produced in each song.

### 2.4 Determining reproductive success and longevity

After locating nests, we monitored nests every 2–3 days. We considered nests successful if we observed adults with fledglings. We also placed thermochrons (iButtons) in nests on nestling day 6 and confirmed success using temperature declines associated with fledging at a reasonable date (~day 10). To determine social parentage, we obtained ~80–200 μl blood samples from the brachial vein of nestlings on day 6 to 7 of the nestling period, and from adults at banding. We stored hematocrit samples in Longmires buffer (Longmire, Gee, Handenkipf, & Mark, 1992) at 4°C until extracting DNA using the DNA IQ^®^ magnetic extraction system (Promega Corp; Madison, WI, USA).

We conducted parentage analysis using five previously described microsatellite loci: Gf01 and Gf12 (Petren, 1998), MME1 (Jeffery, Keller, Arcese, & Bruford, 2001), and Dpμ01 and Dpμ03 (Dawson, Gibbs, Hobson, & Yezerinac, 1997). We used fluorescently labeled primers and ran PCR products on an ABI PRISM 310 Genetic Analyzer^®^ (GMI Inc.; Ramsey, MN, USA) to identify distinct alleles. Procedural details are described in Formica and Tuttle (2009). We determined actual reproductive success of males by adjusting apparent reproductive success for the occurrence of extra‐pair offspring. We assigned parentage to extra‐pair offspring using CERVUS 3.0 (Field Genetics, London, UK; Kalinowski, Taper, & Marshall, 2007), and accepted paternity assignment with 80% certainty or above. Of 474 nestlings, 84 (17.72%) were assigned as extra‐pair offspring. We assigned extra‐pair fathers to 32 (38.10%) of 84 of the extra‐pair offspring.

We determined longevity by noting return of color‐banded males to the breeding population between subsequent seasons. Males in our population show high site fidelity and return to the same, or a close by territory, across multiple years.

### 2.5 Statistical analyses

#### 2.5.1 Preliminary analyses

We used R 2.15.2 (R Core Team, 2012) for the majority of statistical methods. We conducted preliminary analyses using linear models to determine whether playback use, or time and date of recording related to song traits. Song rate increased with playback use (LMM: *t *=* *2.40, β = 0.67 ± 0.28, *p *=* *.02, *N *=* *109 observations, 95 males), when controlling for higher singing rates of white morph males (*t *=* *2.37, β = 0.68 ± 0.28, *p *=* *.02). All other relationships were nonsignificant (*p > *.10). Due to the positive relationship between song rate and playback use, we restricted analyses of the relationship between song rate and fitness to males for which song was solicited.

Preliminary analyses also revealed that a large positive correlation existed between note number and song length (Spearman's correlation: *r *=* *.81, *p *<* *.001). To avoid multicollinearity, we only used note number in subsequent analyses, as this variable is subject to lower measurement error. High and low note frequencies were also correlated (Spearman's correlation: *r *=* *.93, *p *<* *.001), as white‐throated sparrow songs are generally shifted up or down the frequency scale, while maintaining characteristic frequency ratios between notes (Hurly et al., 1991). Thus, we only used high note frequency in statistical models.

#### 2.5.2 Modeling approach

We constructed separate models to evaluate relationships between each of five song traits and fitness variables. These models consisted of the main effects and interactions between morph and (i) average number of notes, (ii) number of triplets produced over three, (iii) song frequency characteristics (2 variables: high note frequency and the deviance in the frequency ratio from the mean), (iv) singing variability (two variables: variability in frequency and in note number), and (v) song rate. We adopted this approach because we were interested in whether relationships between song traits and fitness were morph specific. Testing for morph‐specific relationships required including interactions between morph and song traits in models, and we wanted to avoid constructing models that were too complex, and potentially over fit.

#### 2.5.3 Reproductive success, longevity, and body size

We tested relationships between song traits and lifetime reproductive success using a zero‐inflated generalized linear mixed‐effects model (GLMM) with a negative binomial distribution (R package glmmADMB; Fournier et al., 2012), to account for the high proportion of zeros in our dataset (many males obtained no reproductive success) and overdispersion of the count component. We used first breeding season as a random effect to account for potential variation associated with cohort effects or variation in climatic conditions between years.

We also assessed the relationship between within‐season reproductive success and the five song traits. For note number, the number of triplets over three, singing variability, and song rate, we used reproductive success in the year the song recording was obtained, as these traits could potentially change between seasons depending on physiological state or social interactions. For these models, we used a similar modeling technique as for lifetime reproductive success, but used Poisson rather than negative binomial models, as there was no evidence for overdispersion. We used current rather than first year as a random effect. Song frequency characteristics are stable aspects of the vocal phenotype. Thus, for these traits, we considered within‐year reproductive success across all years of an individual's breeding tenure. We used the same modeling technique as for the other song traits, but with individual identity as an additional random effect to account for multiple observations on the same individual across years.

We used GLMMs with a Poisson distribution to test whether song variables and morph interacted to predict reproductive lifespan, and LMMs to test whether song variables and morph interacted to predict body size (tarsus length; R package lme4; Bates, Maechler, & Bolker, 2012). Models predicting both longevity and body size incorporated first year as a random effect.

#### 2.5.4 Paternity loss within social nests

We examined the relationship between paternity loss within social nests (cuckoldry) and the five song traits using generalized linear mixed‐effects models (R package lme 4; Bates et al., 2012) that incorporated multiple observations on clutches of the same male. We used a binomial model with the number of extra‐pair offspring in the social brood as the response variable and brood size as the binomial denominator. In this model, we entered clutch number as an additional predictor variable, as white‐throated sparrows produce multiple clutches across a breeding season and extra‐pair paternity levels may vary across these clutches. We included individual as a random effect to account for multiple observations on individuals and an observation‐level random effect (clutch ID) to control for overdispersion. We did not analyze the relationship between song traits and success in gaining extra‐pair paternity due to our low rate of paternity assignment.

#### 2.5.5 Morph, individual, and species identity

To determine whether song characteristics differed between individuals or morphs, we used MANOVAs followed by Kruskal–Wallis rank sum tests with a Benjamini–Hochberg correction for multiple comparisons (Benjamini & Hochberg, 1995). We then performed linear discriminant function analyses (DFA) in SPSS 16.0 (SPSS, Chicago, IL, USA) to characterize which aspects of song structure most effectively differentiated individuals or morphs, and determine how effectively these groups could be classified based on song characteristics. For the DFA involving individuals, we used 25 individuals for which we had more than seven high‐quality songs measured. For 5 of the 25 individuals, we had recordings made during more than one recording period, four of which were recorded in multiple years. Songs recorded during different recording periods were not more likely to be classified as belonging to another male than songs made during the same period, so we concluded that song traits were relatively consistent between recording periods and that using songs derived from a single recording period is justifiable. Using songs from a single recording session could reduce variation within songs of individuals, leading to inflation of our ability to correctly identify males based on song traits. However, we were primarily interested in characterizing which aspects of songs strongly differentiated individuals within the population, rather than precisely characterizing rates of classification success. For the DFA involving morph, we used all individuals and averaged song characteristics for each individual. We used Wilks' tests to assess the significance of linear discriminant functions and report loadings of independent variables on each discriminant function. When classifying individuals and the morphs via the discriminant functions, we assigned individuals and morphs an equal prior probability. Using equal prior probabilities prevents disproportionate misclassification of groups (individuals or morphs) that are under‐represented in the sample. We used a chi‐square test to determine whether we were able to predict group membership more often than expected by chance.

In the MANOVAs and DFA, we used the frequency of the first to third notes, the ratios between these notes, the length of the first note, and the number of notes in the song as the song characteristics. We only used the length of the first note, because past research suggests that the length of the first note shows the most variation between individual white‐throated sparrows (Borror & Gunn, 1965). For the analyses involving the morphs, we additionally used measurements of singing variability and the number of triplets produced over three. We used different song traits for these analyses and for the analyses involving fitness because here we were interested in the specifics of song structure, whereas for the analyses involving fitness we were interested in general song performance traits that might reflect fitness differences.

Finally, we calculated the coefficient of variation for the frequency ratio and bandwidth, which are traits previously proposed to function in species recognition (Hurly et al., 1991, 1992). We used *t* tests to evaluate whether these traits differed between song types. Song traits that function in conspecific recognition should be relatively invariable across individuals and song types.

## 3 RESULTS

### 3.1 Diversity in song characteristics

Male white‐throated sparrows displayed four different singing patterns (Figure 1). 46.39% (45/97) of individuals sang a song that ascended in pitch, 44.32% (43/97) sang a descending song, 8.24% (8/97) sang a song that ascended and then descended, and a single individual sang at a constant frequency (monotone, 1.03%). In addition, four males with descending songs also produced an ascending song. In three of these males, the descending song was dominant, with the ascending song nearly always containing a maximum of three notes. In the other male, the descending and ascending song types did not clearly differ in length. For males with two song types, only the frequency characteristics of the descending song type were used in analyses.

All ascending songs displayed a single frequency change between the first and second note. However, descending songs displayed some diversity in frequency shift pattern. One anomalous descending song displayed a single frequency change between the first and second notes. In the remaining 42 descending songs, the major frequency change occurred between the second and third notes. However, in eight songs, the first and second notes were at nearly the same frequency, whereas in the remaining 34 songs there was a small decline in frequency between the first and second notes followed by a larger decline in frequency between the second and third notes (as in Figure 1b). In seven of eight ascending–descending songs, there was an initial frequency increase between the first and second notes followed by a frequency decrease between the second and third notes. However, in one anomalous ascending–descending song, the second and third notes were sung at a nearly constant frequency, followed by a frequency decrease between the third and fourth notes.

Individuals also displayed differences in the types and numbers of notes within their songs. White‐throated sparrows typically sing a series of constant frequency one‐part notes, referred to by Borror and Gunn (1965) as S notes, followed by a series of three‐part notes, or triplets (also referred to as T notes by Borror and Gunn (1965); see Figure 1). 20 (20.6%) of 97 individuals also sang two‐part doublets, either embedded in, or at the end of, their song. In addition, 13 (13.4%) of 97 individuals also sang one note that displayed an initial abrupt upslur and was then constant in pitch (Figure 1a). This type of note conforms to Borror and Gunn's (1965) description of the U note. U notes occurred only in ascending or ascending–descending songs, never in descending songs. Within individuals, different types of notes nearly always occurred in the same order within the song.

The songs of individuals were further distinguished by the absolute frequency at which songs were produced and the bandwidth and frequency ratio between the highest and lowest notes. Table 1 lists information regarding the frequency characteristics of each song type, and allows comparison to an earlier study on the white‐throated sparrow (Hurly et al., 1991).

**Table 1:** Frequency characteristics (Hz) of notes in the four song types observed

	*N*	Mean	Among birds		Within birds	
			*SD*	CV	*SD*	CV
Ascending songs	45 (29, 16)^a^					
Note 1		3,227	474	0.147	38.96	0.012
Note 2		4,090	515	0.126	43.41	0.011
Note 2–1		839	151	0.180	50.97	0.062
Note 2/1		1.264	0.058	0.046	0.011	0.014
Descending songs	42 (26, 16)^b^					
Note 1		4,653	425	0.091	44.62	0.010
Note 2		4,371	536	0.123	37.44	0.008
Note 3		3,476	395	0.114	39.22	0.011
Note 1–2		282	167	0.592	44.41	0.285
Note 1/2		1.069	0.043	0.041	0.010	0.009
Note 2–3		895	189	0.21	45.03	0.076
Note 2/3		1.257	0.041	0.032	0.014	0.011
Ascend/descend	7 (4, 3)^c^					
Note 1		4,272	128	0.030	46.30	0.011
Note 2		5,592	240	0.043	40.15	0.007
Note 3		4,752	73.53	0.015	38.76	0.008
Note 2–1		1,320	187	0.142	50.89	0.040
Note 2/1		1.309	0.043	0.033	0.008	0.011
Note 2–3		840	228	0.271	43.75	0.177
Note 2/3		1.177	0.048	0.040	0.010	0.008
Monotone	1 (1, 0)					
Note 1		3,444			42.87	0.012
Note 1–2		19.15			10.97	1.575
Note 2/1		1.00			0.003	0.003

a *N* = total (white morph males, tan morph males).

b Excludes one descending song in which the largest frequency change occurred between notes 1 and 2.

c Excludes one ascending–descending song in which the largest frequency change occurred between notes 3 and 4.

With respect to performance‐related song traits, average note number ranged from 3 to 8 (mean: 5.20 ± 0.10), the number of triplets produced over three ranged from 0 to 4 (mean: 0.30 ± 0.71), and song rate ranged from 0.20 to 7.40 songs/min (mean: 2.70 ± 0.16). The coefficient of variation averaged across frequency traits ranged from 0 to 0.05 (mean: 0.02 ± 0.001), and the coefficient of variation in note number ranged from 0 to 0.85 (mean: 0.214 ± 0.139).

### 3.2 Reproductive success, longevity, and body size

**Figure 2:**
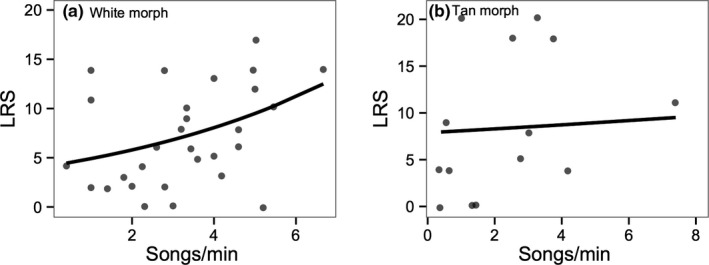
Relationship between lifetime reproductive success (LRS) and song rate within the white (a) and tan (b) morph. Lines show predicted values from the GLMM

Lifetime reproductive success ranged from 0 to 20 offspring (mean: 7.34 ± 0.79) across both morphs, from 0 to 17 (mean: 6.72 ± 0.82) within the white morph, and from 0 to 20 (mean: 8.76 ± 1.79) within the tan morph. Males with higher song rates achieved higher lifetime reproductive success (Table 2a). The interaction between morph and song rate was nonsignificant (*Z *=* *0.95, β = 0.05 ± 0.05, *p *=* *.35). However, the relationship between song rate and lifetime reproductive success was significant within the white morph (Table 2b; Figure 2a) and nonsignificant within the tan morph (Table 2c; Figure 2b). Average note number, the number of triplets produced over 3, song frequency characteristics, and singing variability were all unrelated to lifetime reproductive success (*p *>* *.30 in all cases). Within‐season reproductive success was not related to any song trait (*p > *.10).

**Table 2:** Zero‐inflated negative binomial GLMMs predicting lifetime reproductive success from song rate in response to playback (a) across morphs, (b) within the white morph, (c) in the tan morph

	β ± *SE*	*z*‐Value	*p* > |*z*|
(a) Across morph			
Intercept	1.61 ± 0.24	6.71	<.001
Song rate	0.13 ± 0.06	2.38	.017
Zero inflation	0.15 ± 0.06		
Dispersion parameter	10.02 ± 7.03		
Random effects	*SD*	Variance	*N*
First year	0.42	0.17	15
(b) White morph			
Intercept	1.41 ± 0.28	5.05	<.001
Song rate	0.17 ± 0.07	2.45	.014
Zero inflation	0.12 ± 0.06		
Dispersion parameter	11.89 ± 14.07		
Random effects	*SD*	Variance	*N*
First year	0.36	0.13	13
(c) Tan morph			
Intercept	2.06 ± 0.33	6.23	<.001
Song rate	0.03 ± 0.08	0.31	.76
Zero inflation	0.21 ± 0.11		
Dispersion parameter	>403.43		
Random effects	*SD*	Variance	*N*
First year	0.52	0.27	9

*N *=* *44 total, 30 white, 14 tan.

**Figure 3:**
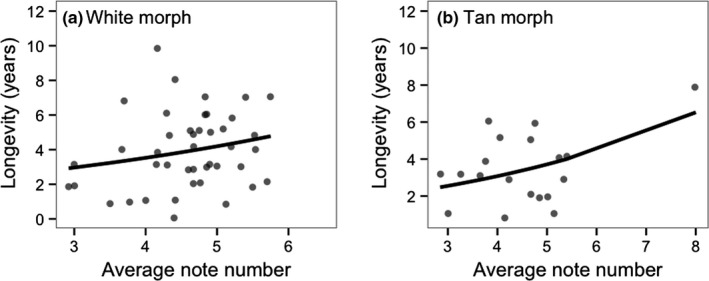
Relationship between breeding longevity and the average number of notes in a male's song in the white (a) and tan (b) morph. Lines show predicted values from the GLMM

Longevity ranged from 1 to 10 years (mean: 4.90 ± 0.15) across morphs, from 1 to 10 (mean: 5.21 ± 0.18) within the white morph, and from 1 to 8 (mean: 4.04 ± 0.22) within the tan morph. Independent of morph, males with more notes in their songs had greater longevity than other males (Poisson GLMM: *Z *=* *2.43, β = 0.15 ± 0.06, *p *=* *.014, *N *=* *62). The interaction between morph and number of notes was nonsignificant (*p *=* *.844). The relationship between number of notes and longevity was nonsignificant but positive in the white morph (*Z *=* *1.45, β = 0.14 ± 0.10, *p *=* *.147, *N = *43; Figure 3a), and significant among tan morph males (*Z *=* *1.96, β = 0.16 ± 0.08, *p *=* *.050, *N *=* *19; Figure 3b). However, as a caveat, the overall relationship between note number and longevity (*p = *.141) and the relationship within the tan morph (*p* = .839) became nonsignificant when removing one tan morph male that sang exceptionally long songs. Results were qualitatively equivalent when using the number of triplets in excess of three (indicative of very long songs) in this model (Poisson GLMM: across morphs: *Z *=* *1.79, β = 0.18 ± 0.10, *p *=* *.072; white morph: *Z *=* *0.74, β = 0.12 ± 0.16, *p *=* *.461; tan morph: *Z *=* *2.22, β = 0.32 ± 0.14, *p *=* *.026). No other song trait was related to longevity (*p *>* *.10).

Body size as measured by tarsus length was not related to song frequency characteristics, or to any other song trait (LMM: *p *>* *.10 in all cases, *N *=* *81).

### 3.3 Paternity loss within social nests

White morph males had more extra‐pair offspring in their social nests when compared to tan morph males (GLM, binomial family: *Z *=* *−3.74, β = −3.06 ± 0.82, *p *<* *.001, *N *=* *221). However, no song trait showed a statistically significant relationship to levels of paternity loss (*p > *.10).

### 3.4 Individual, morph, and species identity

#### 3.4.1 Song differences between individuals

**Figure 4:**
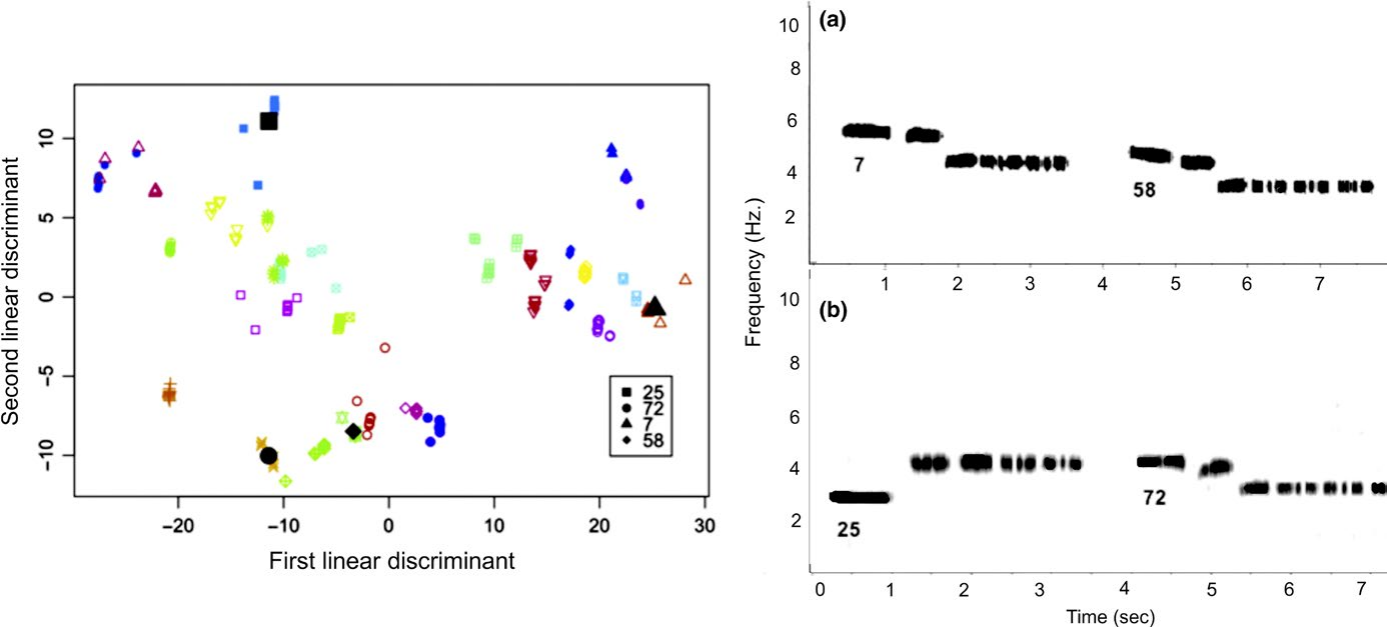
Separation of individuals on the first and second linear discriminant functions derived from song characteristics. Centroids are shown for four representative individuals, whose songs are shown in the spectrograms. The first linear discriminant separates individuals largely according to song frequency, as illustrated in spectrogram a. Males toward the left of the plot sing at high frequency like individual 7, whereas males to the right sing lower, like individual 58. The second linear discriminant separates individuals largely by song pattern, as illustrated by spectrogram b. Thus, birds on the top of the plot display ascending songs like male 25, whereas birds on the bottom of the plot show descending songs like males 72, 7, and 58. Note that males 58 and 72 have the most similar song pattern, with the second note slightly lower than the first, whereas male 7 has the first two notes almost equal in frequency

Individuals had distinctive songs (MANOVA: *F*_5,456_ = 11.13, *p *<* *.001), with individuals differing significantly in terms of all of the song characteristics examined (Kruskal–Wallis tests: *p *<* *.009 in all cases). The discriminant function analysis extracted seven significant linear discriminant functions, with the first discriminant function describing 81.5% of total variance in song traits, and the second discriminant function describing an additional 11.0% (Table 3a). The frequency of the notes displayed high loadings on the first discriminant function, suggesting that singing frequency differentiated more individuals than ratios between notes, note number, or the length of note 1 (Table 3b). The second discriminant function displayed a high loading for the ratio between the first and second notes (Table 3b). Thus, the second discriminant function appears strongly related to song type, in that it distinguishes song pattern. In essence, many individual white‐throated sparrows share the same basic singing pattern, and within a song type, frequency of singing distinguishes individuals (first linear discriminant). This can be visualized by plotting individual centroids on the first and second discriminant functions (Figure 4). Of the song characteristics, the number of notes in the song was the least strongly related to individual identity, as indicated by the fact that note number loaded onto the seventh discriminant function.

**Table 3:** Results from the DFA regarding individual identity. (a) Variance described and significance tests for the discriminant function. (b) Variable loadings

(a)				
Function	Eigenvalue	% Variance	Cumulative %	Canonical corr.
1	275.991	81.5	81.5	.998
2	37.096	11.0	92.4	.987
3	9.952	2.9	95.4	.953
4	8.398	2.5	97.9	.945
5	4.382	1.3	99.1	.902
6	2.163	0.6	99.8	.827
7	0.736	0.2	100.0	.651

	Wilks' lambda	Chi‐square	*df*	*p*
1 through 7	<.001	4061.820	168	<.001
2 through 7	<.001	2740.184	138	<.001
3 through 7	<.001	1884.757	110	<.001
4 through 7	.004	1322.282	84	<.001
5 through 7	.034	795.771	60	<.001
6 through 7	.182	400.257	38	<.001
7	.576	129.651	18	<.001

(b)	Function						
	1	2	3	4	5	6	7
Frequency note 2	0.838^a^	0.374	−0.107	−0.145	−0.190	−0.275	−0.120
Ratio note 1–2	−0.038	0.650^a^	0.117	−0.296	−0.122	0.582	−0.347
Length note 1	−0.031	0.200	−0.512	−0.075	0.796^a^	0.238	0.028
Frequency note 3	0.160	0.368	0.444	−0.260	0.165	0.672^a^	−0.310
Ratio note 2–3	−0.087	0.312	0.543	−0.171	0.277	0.653^a^	−0.261
Frequency note 1	0.243	−0.488	−0.271	0.431	−0.039	−0.525^a^	0.408
Number of notes	0.003	−0.004	0.016	−0.075	−0.055	−0.099	0.991^a^

a Indicates the highest loading of each variable on a discriminate function.

The discriminant function analysis classified 93.3% of songs (235 of 252 songs from 25 males) as belonging to the correct male, whereas by chance alone, only ~4.0% (1/25) of songs would be classed correctly. The increase in classification success allowed by the DFA was significant ($\chi_{1}^{2}=5227.865$, *p *<* *.001). Cases of misclassification involved males that shared both a similar singing frequency and song type.

#### 3.4.2 Song differences between the morphs

The morphs differed significantly with respect to the song characteristics measured (MANOVA: *F*_11,81_ = 3.05, *p *=* *.002). Kruskal–Wallis tests indicated that the morphs tended to differ in the coefficient of variation in frequency traits ($\chi_{1}^{2}=6.24$, *p = *.054), the length of the first note ($\chi_{1}^{2}=6.99$, *p *=* *.054), and the number of triples produced above three ($\chi_{1}^{2}=4.81$, *p *=* *.084). The other song characteristics, notably including absolute frequencies and the ratio between notes, did not differ between the morphs (*p *>* *.20).

The DFA extracted a significant first linear discriminant (Wilks' lambda = .751, $\chi_{10}^{2}=24.676$, *p *=* *.006, canonical correlation = .499), although the eigenvalue (0.332) associated with this discriminant was small, indicating that much variation in song structure is not related to morph. At the group centroid, white morph males had a value of −0.403 on the linear discriminant, whereas tan morph males had a value of 0.806. Song frequency variability, length of the first note, and number of triplets sung above three showed relatively high positive loadings on the linear discriminant, whereas average number of notes showed a relatively high, negative loading (Table 4). Tan morph males had higher values on the linear discriminant than white morph males, suggesting that tan morph males display more variable songs, longer first notes, and sometimes produce exceptionally long songs (sing more than three triplets) while at the same time tending to sing fewer notes on average. The frequency of notes and ratios between note frequencies showed comparatively little relationship to the linear discriminant, and thus morph identity.

**Table 4:** Variable loadings from the DFA regarding morph identity

Variable	Loading
Variability (freq.)	0.513
Length note 1	0.505
Triplets above 3	0.431
Average note number	−0.319
Variability (note number)	0.202
Frequency note 3	0.175
Frequency note 2	0.170
Frequency note 1	0.071
Ratio note 1–2	−0.039
Ratio note 2–3	−0.006

DFA successfully classified 71.0% of males (66 of 93) to the correct morph based on song characteristics. 47 of 62 white morph males (75.8%) and 19 of 31 (61.3%) tan morph males were classed correctly. Four tan morph males were not used in the analysis because a low number of songs were measured. If males were randomly classed into groups based on the 50% prior probabilities used in the analysis, only ~50% of males would be classed correctly. Thus, the DFA led to a 21.0% increase in classification success. A chi‐square test indicated that this increase in classification success is statistically significant ($\chi_{1}^{2}$ = 16.35, *p *<* *.001).

### 3.5 Variation in proposed species identity song traits: frequency ratio and bandwidth

For ascending songs, the coefficient of variation in the bandwidth between notes 2 and 1 was greater than the coefficient of variation in the frequency ratio between these notes (Table 1), and this was also true in the case of descending songs when comparing the bandwidth and frequency ratios for notes 2 and 3 (Table 1). The mean frequency ratios that we observed for ascending (1.264) and descending (1.257) songs are close to those reported by Hurly et al. (1991), who reported mean frequency ratios of 1.237 and 1.281 for ascending and descending songs, respectively.

Nevertheless, the two rare song types that we observed, ascending–descending and monotone, differ in their frequency characteristics relative to the more common song types. Monotone has an atypical frequency ratio of 1. The ascending–descending song type tended to have a larger frequency ratio between notes 2 and 1 than ascending songs (*t* test: *t*_13_ **= 2.094, *p *=* *.056, *N *=* *53), and had a lower frequency ratio between notes 2 and 3 than descending songs (*t* test: *t*_8_ **= −3.905, *p *=* *.004, *N *=* *50). Interestingly, for ascending–descending songs, the coefficient of variation for absolute frequency was as low as that for the frequency ratio, in part reflecting the fact that all individuals with ascending–descending songs sang at relatively high frequencies.

## 4 DISCUSSION

Our study suggests that the white‐throated sparrow's song has evolved to fulfill multifaceted signaling functions and that different song traits convey distinct messages. First, correlations between song rate and lifetime reproductive success, and note number and longevity, suggest that these song traits communicate information about fitness. Furthermore, the stronger correlation between song rate and reproductive success within white morph males provides some indication that song traits are under stronger sexual selection in the more polygynous white morph. Second, individually distinctive song traits, particularly the frequency at which individuals sing, may have diversified through negative frequency‐dependent selection for advertising individual identity. Third, the existence of song traits allowing for morph discrimination suggests that certain song traits can indicate morph identity. Finally, our study offers insight into the variability of a potential species identity trait, the frequency ratio between notes. In sum, our study suggests that when a white‐throated sparrow sings, receivers can simultaneously decode information regarding male fitness, morph, and individual and species identity.

Two performance‐related song traits were related to fitness metrics and thus may be sexually selected indicator traits. First, song rate in response to playback was related to lifetime reproductive success. Song rate may reliably indicate male quality, and hence be subject to sexual selection, because only males in good condition are able to sustain high rates of song output without suffering physiological costs (Nowicki & Searcy, 2004). Higher‐quality males may also display stronger responses to a territorial intrusion (i.e., sing at higher rates in response to playback) if they are better able to cope with costs of engaging in antagonistic encounters. Past studies have linked song rate to an array of indicators of male quality including body condition, female choice, parental care, and fitness proxies (Murphy et al., 2008; Otter, Chruszcz, & Ratcliffe, 1997; Welling et al., 1997). For instance, European starlings (*Sturnus vulgaris*) that sing longer song bouts pair earlier and are preferred by females in a laboratory setting (Eens, Pixten, & Verheyen, 1991) and male song sparrows (*Melospiza melodia*) that sing at higher rates display higher body mass and produce heavier nestlings (Grunst & Grunst, 2014). However, few previous studies have linked song rate to metrics of reproductive output. Notably, there was no relationship between any song trait and within‐season reproductive success. Reproductive success within seasons may be stochastic such that lifetime measurement of reproductive output is necessary to detect relationships with song traits.

Second, both average note number and the number of triplets in excess of three in a male's song correlated with longevity. Songs with many notes and triplets may be more challenging to produce and thus reliably indicate male quality or condition, and predict longevity. The number of notes in a male's song could also increase lifespan by facilitating access to resources or territories. In support of this possibility, past studies have related song length to male fighting ability and aggressive motivation (Lattin & Ritchison, 2009; Linhart, Slabbekoorn, & Fuchs, 2012).

As a caveat, although the frequency characteristics of the songs sung by individual males are repeatable, as established by our discriminant function analysis, we were not able to establish the repeatability of singing rate or song length (as reflected by note number), because we had multiple recordings for relatively few males. Selection can act only on repeatable traits. Thus, testing for repeatability will be a necessary next step to establishing the relationship between these performance‐based song traits, fitness, and selection. Obtaining longer recordings to assess singing rate might also be desirable. However, our metric of singing rate represents the immediate vocal response to a territorial intrusion, which in some cases has been found to more strongly relate to fitness than spontaneous song rate (Cain, Cockburn, & Langmore, 2015).

We predicted that relationships between song traits and fitness would be stronger within promiscuous white morph males, consistent with stronger sexual selection on song traits within the white morph. There was not a statistically significant interaction between male morph and song traits in predicting reproductive success. However, the relationship between lifetime reproductive success and song rate was only significant within the white morph and was nonsignificant within tan morph males. Contrary to predictions, the relationship between note number and longevity was stronger within the tan morph. However, this relationship was largely driven by one tan morph male, and when removed, there was no relationship between longevity and fitness in either morph. Overall, these results offer some indication that, as predicted, sexual selection on song traits may be stronger within white morph males than within tan morph males. However, our sample size of tan morph males was smaller due to lower singing rates in this morph, which does limit our ability to test for morph differences.

In contrast to some past research in other species (Araya‐Ajoy, Chaves‐Campos, Kalko, & DeWoody, 2009; Nemeth et al., 2012; Seddon, Amos, Mulder, & Tobias, 2004), we found no indication that the frequency characteristics of white‐throated sparrow song are related to fitness. Frequency characteristics were also unrelated to morph. Rather, as discussed further below, the frequency characteristics of songs appear to function in signaling individual identity. Theory on the evolution of individual identity signals suggests that these signals should be inexpensive to produce, and condition independent, in contrast to predictions for sexually selected signals (Tibbetts & Curtis, 2007; Tibbetts & Dale, 2007). Thus, given that song frequency is an individual identity trait, the finding that frequency characteristics are not related to fitness is consistent with theory, and supports a multiple messages signaling model, in which different song traits may communicate discrete types of information (Hebets & Papaj, 2005; Møller & Pomiankowski, 1993; Rivera‐Gutierrez et al., 2010).

Also consistent with song frequency playing a discrete role in signaling individual identity, song frequency was unrelated to body size (Hall, Kingma, & Peters, 2013; Price, Earnshaw, & Webster, 2006). This contrasts with some past work in avian species. For instance, larger purple‐crowned fairy‐wren (*Malurus coronatus coronatus*) males sing advertising songs containing low‐pitched notes (Hall et al., 2013). However, other research has failed to find a relationship between song frequency and body size, particularly in oscine birds (Cardoso et al., 2008; Galeotti, Saino, Sacchi, & Møller, 1997; Irwin, Thimgan, & Irwin, 2008; Logue et al., 2007). This may be because the sophisticated vocal systems of oscines give them the capacity to perform over a wide frequency range (Cardoso et al., 2008).

The results of our discriminate function analysis support previous research suggesting that a primary function of white‐throated sparrow song is signaling individual identity, and specifically demonstrate the importance of song frequency as an individual identity trait. The first linear discriminant was primarily related to the frequency at which individuals sing, suggesting that negative frequency‐dependent selection may act to promote diversity in this song trait. The frequency at which males sing may be analogous to other traits that display high levels of individual variation, and signal individual identity, such as plumage color in ruffs (Dale et al., 2001) and facial coloration in brown paper wasps (Tibbetts, 2002). The second discriminant function was related to the ratio between the frequency of the notes, and thus song type. However, the second discriminant function was associated with a much lower amount of variation than the first (11% vs. 81%). Song type may be under stabilizing selection in favor of population typical singing patterns. As a result, many white‐throated sparrows share the same basic, population typical song types and variation in singing frequency within these song types plays the strongest role in distinguishing individuals. We achieved very high success in classifying the songs of individual birds (93.3%), which indicates the great potential of song as an individual identity trait. The success of this classification may have been even further improved if we had incorporated additional complexities into the analysis, such as variation in the types and ordering of notes represented in the songs of different individuals. Past work has demonstrated that white‐throated sparrows recognize the songs of territorial neighbors, suggesting that signaling individual identity through song plays an important role in mediating territorial interactions (Brooks & Falls, 1975a,1975b).

Results also establish the white‐throated sparrow as a rare example of a species in which the vocal phenotype can communicate information about morph. Consistent with past research suggesting structurally similar songs in the two morphs (Hurly et al., 1991; Lowther, 1961), the morphs shared the same basic song types and frequency characteristics, but were separated by subtle differences in song production. Morph‐specific song traits included longer first notes and higher song variability in tan morph males, and a tendency for white morph males to sing more notes. In addition, despite tending to sing fewer notes on average, tan morph males were more likely to produce very long songs containing triplets in excess of three. Remarkably, one tan morph male was recorded singing a song with 11 notes and 7 triplets. High singing variability and production of very long songs by tan morph males could serve to signal morph identity, but could also reflect poorer canalization of song production. Indeed, larger song‐specific brain regions could facilitate more consistent song production in white morph males (DeVoogd, Houtman, & Falls, 1995), and consistent singing in white morph males could be favored by sexual selection. However, we found no relationship between singing consistency and fitness, even in the white morph. By signaling their morph when singing, tan morph males could decrease aggression from white morph males and attract mates of the appropriate morph. Playback experiments could be employed to test this hypothesis.

Our study was consistent with Hurly et al. (1991) in indicating that variation in the frequency ratio between notes is lower than variation in bandwidth. This result supports the suggestion that the frequency ratio, rather than bandwidth, may function in conspecific recognition. Furthermore, the fact that our estimates for the frequency ratios in ascending and descending songs are very close to those reported by Hurly et al. (1991) for a population of white‐throated sparrows breeding in Algonquin Park, Ontario, is intriguing, and suggests that these ratios may be a stable characteristic of the species that spans across populations. On the other hand, the rare ascending–descending and monotone song types observed in our population differed in the frequency ratio when compared to the more common song types, suggesting that scope for variation in this trait does exist. These rare song types may be more common in other populations. Indeed, Borror and Gunn's (1965) tabulation of variation in white‐throated sparrow song included a number of song types that appear to have a different frequency ratio than ~1.2, including the monotone type song. More work spanning a larger geographic scale is needed to decipher the full range of variation that exists in the frequency ratio of white‐throated sparrow song.

In addition, although Hurly et al. (1990, 1992) demonstrated a greater response to playback of white‐throated sparrow songs with the population typical frequency ratio, independent of bandwidth, it is not clear that deviation from a typical ratio affects fitness. Indeed, in our study, we found no indication that deviation from the mean frequency ratio was related to fitness variables. This may be because of the inherently low variation in the frequency ratio. However, we did have two individuals in our sample that had frequency ratios that fell more than 3.8 standard deviations from the mean, and which would thus have been classified by Hurly et al. (1990) as aberrant songs. An additional seven individuals had frequency ratios that fell nearly two standard deviations from the mean.

In summary, our study suggests that some performance‐related song traits (song rate and length) in the white‐throated sparrow are related to fitness, with some indication that sexual selection on song traits is stronger within the more polygynous white morph. Moreover, song traits provide enough information to predict individual and morph identity, suggesting that alternative selective forces have acted on different song characteristics to promote distinct signaling functions. Signaling individual and morph identity may facilitate adaptive social interactions, including territorial encounters and social pair formation. Finally, the frequency ratio between notes may function in conspecific recognition, although additional work is needed to assess the potential for diversity in white‐throated sparrow singing patterns. Thus, the white‐throated sparrow's song is an auditory signal that has evolved to communicate multiple messages regarding the male's phenotype.

## CONFLICT OF INTEREST

None declared.

## AUTHOR CONTRIBUTIONS

MLG analyzed song recordings, conducted statistical analyses, and wrote the manuscript. ASG conducted statistical analyses and wrote and revised portions of the manuscript. VAF conducted song recordings, contributed to study design, and helped in revising the manuscript. EMT and RAG designed and funded the study, and aided in the data collection, analysis, and writing process.
